# An Ostomy Self-management Telehealth Intervention for Cancer Survivors: Technology-Related Findings From a Randomized Controlled Trial

**DOI:** 10.2196/26545

**Published:** 2021-09-27

**Authors:** Ronald S Weinstein, Michael J Holcomb, Julia Mo, Peter Yonsetto, Octavio Bojorquez, Marcia Grant, Christopher S Wendel, Nancy J Tallman, Elizabeth Ercolano, Zuleyha Cidav, Mark C Hornbrook, Virginia Sun, Ruth McCorkle, Robert S Krouse

**Affiliations:** 1 Arizona Telemedicine Program The University of Arizona Health Sciences Tucson, AZ United States; 2 Hospital of the University of Pennsylvania Philadelphia, PA United States; 3 Nursing Research and Education City of Hope National Medical Center Duarte, CA United States; 4 The University of Arizona Tucson, AZ United States; 5 School of Public Health Yale University New Haven, CT United States; 6 University of Pennsylvania Philadelphia, PA United States; 7 Center for Health Research Kaiser Permanente, Northwest Region Portland, OR United States; 8 Yale University New Haven, CT United States; 9 Department of Surgery Perelman School of Medicine University of Pennsylvania Philadelphia, PA United States

**Keywords:** telehealth, telemedicine, cloud computing, ostomy, cancer survivors, family caregivers, self-management, patient education, videoconferencing, mobile phone

## Abstract

**Background:**

An Ostomy Self-management Telehealth (OSMT) intervention by nurse educators and peer ostomates can equip new ostomates with critical knowledge regarding ostomy care. A telehealth technology assessment aim was to measure telehealth engineer support requirements for telehealth technology–related (TTR) incidents encountered during OSMT intervention sessions held via a secure cloud-based videoconferencing service, *Zoom for Healthcare*.

**Objective:**

This paper examines technology-related challenges, issues, and opportunities encountered in the use of telehealth in a randomized controlled trial intervention for cancer survivors living with a permanent ostomy.

**Methods:**

The Arizona Telemedicine Program provided telehealth engineering support for 105 OSMT sessions, scheduled for 90 to 120 minutes each, over a 2-year period. The OSMT groups included up to 15 participants, comprising 4-6 ostomates, 4-6 peer ostomates, 2 nurse educators, and 1 telehealth engineer. OSMT-session TTR incidents were recorded contemporaneously in detailed notes by the research staff. TTR incidents were categorized and tallied.

**Results:**

A total of 97.1% (102/105) OSMT sessions were completed as scheduled. In total, 3 OSMT sessions were not held owing to non–technology-related reasons. Of the 93 ostomates who participated in OSMT sessions, 80 (86%) completed their OSMT curriculum. TTR incidents occurred in 36.3% (37/102) of the completed sessions with varying disruptive impacts. No sessions were canceled or rescheduled because of TTR incidents. Disruptions from TTR incidents were minimized by following the TTR incident prevention and incident response plans.

**Conclusions:**

Telehealth videoconferencing technology can enable ostomates to participate in ostomy self-management education by incorporating dedicated telehealth engineering support. Potentially, OSMT greatly expands the availability of ostomy self-management education for new ostomates.

**Trial Registration:**

ClinicalTrials.gov NCT02974634; https://clinicaltrials.gov/ct2/show/NCT02974634

## Introduction

### Background

An intestinal stoma, or ostomy, is a surgically created opening in the abdomen that provides an alternate pathway for stool or urine to exit the body. An ostomy may be needed for a patient due to cancer, trauma, inflammatory bowel disease, bowel obstruction, infection, incontinence, or diverticular disease [1]. According to the United Ostomy Associations of America, approximately 100,000 ostomy surgeries are performed annually in the United States [2]. Ostomies have been shown to be associated with multiple health-related quality of life difficulties, irrespective of the type of or reason for ostomy [3]. Ostomy complication rates of 21%-70% have been reported in previous studies [4].

Successfully caring for and living with an ostomy requires the development of specific skills and regimens by the patient. Without proper care, ostomy sites may develop irritation or infection. Ostomates may develop psychosocial complications if they lack knowledge of ostomy self-management or coping skills. Many ostomates face multiple barriers to support and resources, such as travel distances, lack of transportation, financial restrictions, and lack of access to certified wound, ostomy, and continence nurses (WOCNs) [5].

A systematic review conducted by Faury et al [6] found that patient education programs have a positive impact on certain psychosocial and self-management skills for colorectal cancer survivors with ostomies. However, no consensus has been reached regarding the optimal curriculum content and delivery method. Our previous studies have examined the unique patient-reported challenges faced by colorectal cancer survivors with ostomies, highlighting the importance of education, skill building, and emotional support (both formal and informal) [7]. It is critical to develop accessible survivor education programs that address the needs, concerns, and struggles experienced by cancer survivors with ostomies.

### Objectives

We developed the Ostomy Self-management Telehealth (OSMT) intervention to help ostomates learn to care for their ostomy sites and adapt to living with an ostomy. The program content was first delivered in an in-person setting in which WOCN educators taught new ostomates about ostomies, ostomy care, and life with an ostomy. The curriculum design provided an opportunity for new ostomates to learn from people with long-term ostomies (peer ostomates) who have successfully adapted to life with an ostomy [5,7,8]. While this program was beneficial for cancer survivors with ostomies, survivors had barriers to participation [8]. Programs that deliver education to patients with advanced disease may face participant attrition due to disease progression and other limitations that would prompt withdrawal from the program [9]. Technology-aided alternatives such as videotaped education or computer-aided instruction provide accessibility but sacrifice the interactivity and active discussion offered by in-person education sessions [10].

Telehealth is potentially a preferred alternative for delivering self-management education to patients with chronic diseases [11,12]. However, patients face potential technology barriers as well, including challenges related to their preparedness to participate in videoconferencing, suitability of various types of videoconferencing devices, and local network connectivity management issues [13-17]. This paper aims to examine technology challenges, issues, and opportunities encountered in the use of telehealth in a cancer survivor education program [12-15].

## Methods

### Recruitment and Design of the OSMT Intervention

The overall design of the OSMT intervention, including the block randomization method, has been described in detail elsewhere [5]. The OSMT program is a multisite group intervention designed to support self-efficacy and patient activation for ostomy self-management among cancer survivors. This study was approved by the Human Subjects Protection institutional review boards of the participating research sites. The OSMT program was delivered to 21 consecutive groups of ostomates via a series of 4 telehealth sessions conducted by a team of trained WOCN educators and peer ostomates (hereafter referred to as *peers*). A fifth group education session that ostomates did not attend was delivered via telehealth to family caregivers (FCs) or support persons (SPs). OSMT sessions typically lasted 90-120 minutes and were generally held weekly. Study participants were recruited from health care delivery sites in 3 cities: City of Hope National Medical Center in Los Angeles, California; the Hospital of the University of Pennsylvania in Philadelphia, Pennsylvania; and Yale-New Haven Medical Center in New Haven, Connecticut. This study enrolled cancer survivors who were English-speaking, aged ≥21 years, and underwent ostomy surgery at least 6 weeks before the first session. FCs or SPs aged ≥21 years who provided assistive care for the patients were also enrolled, although their participation was not a requirement for patient enrollment. Participants were randomized to either the intervention arm or the usual care (UC) arm. Participants in both arms were given information postoperatively on ostomy-related resources in their respective areas; however, the study team did not provide UC arm participants with the OSMT program.

### Telehealth Design, Preintervention Preparations, and Technological Support for Participants

The OSMT program used telehealth technology managed and supported by the Arizona Telemedicine Program (ATP) at the University of Arizona’s College of Medicine, in Tucson, Arizona [5,14,15]. OSMT sessions with groups of ostomates were held via the *Zoom for Healthcare* encrypted videoconferencing service provided by Zoom Video Communications Inc. The *Zoom for Healthcare* service enables secure group video calls (SGVCs). A Health Insurance Portability and Accountability Act business associate agreement between the University of Arizona’s ATP and Zoom Video Communications was executed as required.

In this OSMT intervention, the OSMT trainee groups were linked into SGVCs as *virtual training* groups. Before the first session of the OSMT curriculum for each OSMT group, study coordinators from each accrual site paired up with the ATP telehealth engineers to train participants on the technologies they would use to participate in the SGVCs. ATP implemented telehealth technology incident prevention plans (IPPs) and incident response plans (IRPs). IPPs included instructing, testing, and requesting participants to connect up to 30 minutes early for the scheduled OSMT sessions to allow time to resolve any last-minute technical difficulties before the start of the session and after telehealth technology–related (TTR) incident follow-up to ensure that any problems were fully resolved. Instruction was provided to ostomates and respective FCs or SPs on loading the Zoom (hereafter referred to as videoconferencing software) app on their engagement device of choice (a PC, laptop, smartphone, or tablet), on using basic videoconferencing software controls, and on following privacy and SGVC etiquette during the OSMT sessions. These instructions and pre-OSMT-session test video calls were designed to familiarize all participants with commonly used videoconferencing software features, such as muting and unmuting the microphone, enabling and disabling sharing of their video camera’s image, and the positioning of their video camera. If participants did not have an engagement device or an internet connection, a study tablet was loaned to them. The IRPs were uncomplicated. ATP telehealth engineers staffed each OSMT session in-person to provide an immediate response to any TTR incidents that occurred. In cases where participants encountered technology difficulties (problems with network connectivity or videoconferencing devices) or needed additional assistance with the videoconferencing software platform, ATP telehealth engineers could immediately and often proactively provide technical support as needed. Ostomates and their respective FC or SP generally participated in OSMT sessions from home but could also optionally connect from an alternative location with adequate privacy and broadband internet connectivity.

PCs and laptops were the most common types of devices used by intervention patients to connect to OSMT sessions, followed by tablets and smartphones (Table 1). Most patients used a single device for all of their OSMT sessions.

**Table 1:** Devices used to connect to Ostomy Self-management Telehealth (OSMT) sessions by 93 intervention patients that participated in OSMT sessions.

Device	Values, n (%)
PC or laptop	45 (48)
Tablet or iPad (Apple Inc)	26 (28)
Smartphone	11 (12)
Unknown	10 (11)
More than 1 device	1 (1)

### Ostomy Self-management Telehealth SGVCs

SGVCs included up to 15 participants per session, including up to 6 ostomates, 6 peers, 2 WOCN educators, and an ATP telehealth engineer. Generally, 2 WOCN educators staffed each OSMT session. This paradigm was not intended for tandem-teaching; rather, 1 WOCN educator was responsible for teaching all 4 patient-training units and the other taught the FC and SP training unit. In addition, 4-6 peers were integral to the delivery of the curriculum and discussion. Each WOCN educator provided backup and support to the other for the OSMT sessions as needed. Session disruptions for any reason during multiparticipant OSMT sessions were of concern because they are distracting and waste multiple participants’ time simultaneously. The built-in redundancy of WOCN educators and peers helped alleviate such occurrences.

New cohorts of ostomates for the OSMT program were created on a rolling basis as new ostomates accrued to the intervention arm of the study. The pair of WOCN educators connected to all group sessions from a Health Insurance Portability and Accountability Act–compliant videoconferencing room in the ATP office suite on the first floor of the Arizona Health Sciences Library, located at the University of Arizona campus in Tucson [14]. In general, peers participated from their homes.

### Telehealth Support Operations

At the ATP headquarters in Tucson, Arizona, the ATP engineer on duty joined the 2 nurse educators in the ATP videoconferencing room for each OSMT session. This setup allowed for expedited technical assistance and troubleshooting by the ATP telehealth engineer during the sessions. During each OSMT session, the engineer responded immediately to any technical problems (eg, loss of audio or telecommunications network instability). For complex support incidents requiring more than a few minutes to rectify, the engineer would physically relocate offline to the ATP’s Control Room on the same floor as the ATP videoconference room, approximately 50 feet away, and work with the participants experiencing TTR problems to resolve the problem and assist them in reconnecting to their session. The engineer then returned to the videoconferencing room with the WOCNs to continue monitoring and supporting the ongoing session.

The ATP telehealth engineers each had decades of experience working in the telehealth industry. They had extensive prior experience working with rural telehealth site coordinators, nurses, and the public, but had little prior experience working directly with patients.

Based on the study protocol and participant agreement, OSMT sessions were not recorded in the interest of preserving the privacy and confidentiality of the ostomates and FCs or SPs participating in each session. In the United States, it is standard practice in most clinical telehealth applications not to record videoconferencing sessions with patients.

### Outcomes and Data Collection

Throughout this study, data on telehealth support, participant acceptance, technology-associated problems, and related outcomes were collected using several approaches. TTR observations from WOCN, peer, and telehealth engineer field notes were stored in the form of contemporaneous notes in the master data set of the OSMT-session data. The comments also included non-TTR observations.

The observations were classified into 2 categories. A *major* incident involved telehealth engineer intervention. A *minor* incident did not require telehealth engineer intervention and could be resolved by an ostomate, a peer, or a WOCN nurse educator. The field notes collected were reviewed to identify and count the TTR incidents experienced in each session. Study coordinators collected the session data and field notes recorded by the OSMT program team and stored them in a study database that contained all OSMT-session written observations. In addition, reasons for declining study participation owing to technical problems or attrition due to technical problems were also recorded as part of the study implementation data.

### Data Analysis

All comments containing TTR observations were analyzed by an ATP telehealth engineer to identify TTR support incidents and to determine whether an engineer intervened for each incident. Each TTR incident was counted as major if an engineer intervened and minor if an engineer did not intervene. TTR incidents were also categorized by audio, video, internet connection, software, or equipment problems. TTR incidents with no details about the nature of the problem were categorized as *unspecified.* Specific search terms were used to identify technology-related concepts and comments for analysis purposes (Textbox 1). All categorized data were reviewed by the research team. Data that were discordantly categorized were discussed by the research team, and a consensus decision was made. Counts for all groups and categories of TTR incidents were tallied using descriptive summary statistics (frequencies, percentages, etc).

**Technology search terms.**

**Search Terms**

- *Android*
- *App*
- *Audio*
- *Broadband*
- *Call* (nonspecific, but useful, “technology term” identifiers)
- *Camera*
- *Cellular*
- *Computer*
- *Connect(ed)*
- *Connection*
- *Data*
- *Device*
- *Disconnect(ed)*
- *Display* (nonspecific, but useful, “technology term” identifiers)
- *Drop(ped)*
- *Hear* (nonspecific, but useful, “technology term” identifiers)
- *Install*
- *Internet*
- *iOS*
- *iPad*
- *iPhone*
- *Laptop*
- *Light(ing)* (nonspecific, but useful, “technology term” identifiers)
- *Mic* (nonspecific, but useful, “technology term” identifiers)
- *Microphone*
- *Mute* (nonspecific, but useful, “technology term” identifiers)
- *Network*
- *Phone*
- *Screen*
- *See* (nonspecific, but useful, “technology term” identifiers)
- *Signal*
- *Smartphone*
- *Tablet*
- *Technical*
- *Technology*
- *Telehealth*
- *Telemedicine*
- *Troubleshooting*
- *Uninstall*
- *Unmute*
- *Video*
- *WiFi*
- *Wired* (nonspecific, but useful, “technology term” identifiers)
- *Wireless*
- *Zoom*

## Results

### Study Participation, Completion, and Telehealth Use

**Figure 1:**
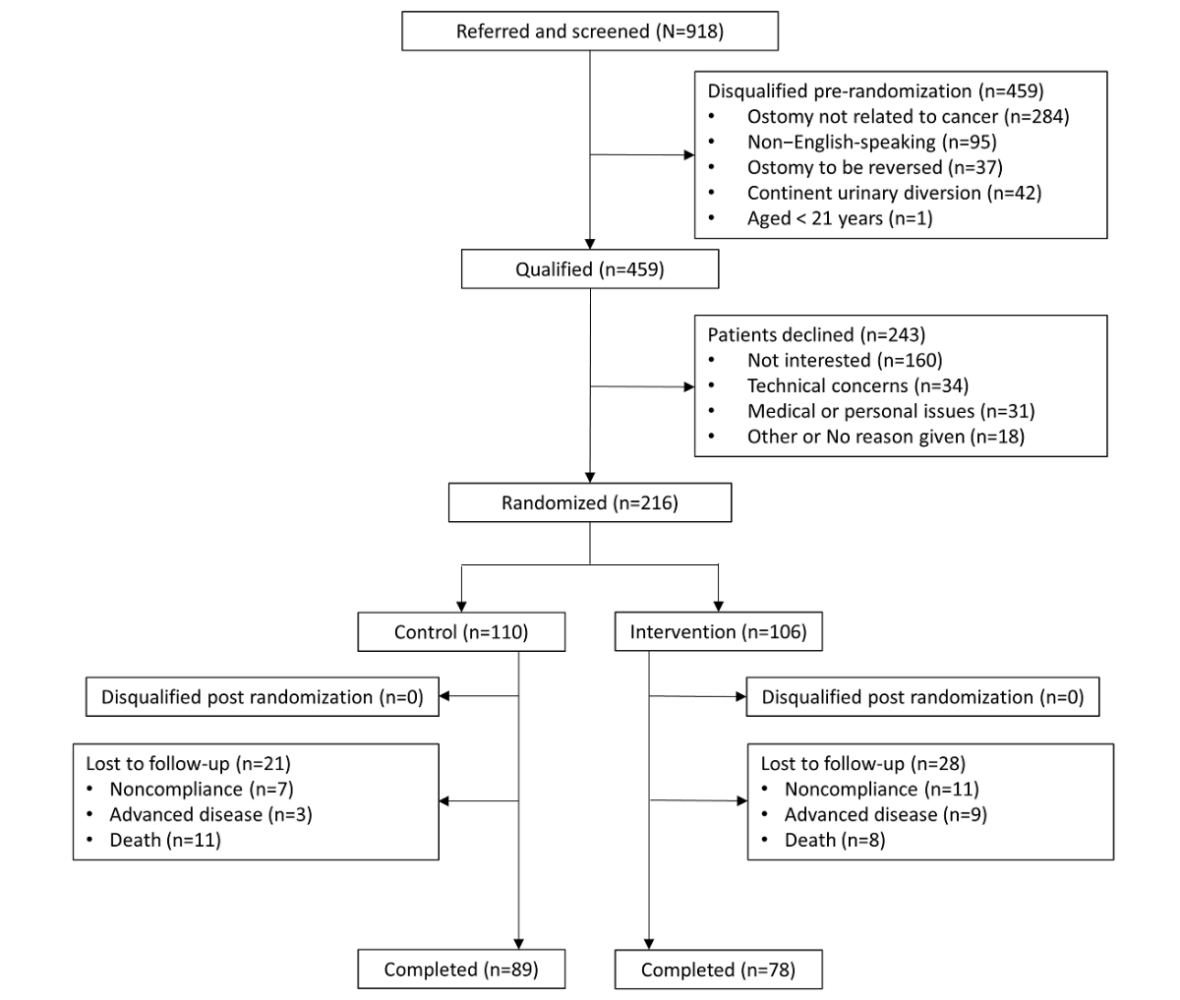
CONSORT (Consolidated Standards of Reporting Trials) flow diagram.

The study CONSORT (Consolidated Standards of Reporting Trials) flow diagram [18] showing recruitment and retention is presented in Figure 1. Of the 459 cancer survivors qualified for participation in the study, 7.4% (34) declined participation due to technology-related concerns. Of the 34 technology-related concerns among qualified patients who declined participation, 88% (30) were related to *fear of technology*, and 12% (4) claimed to have no prior experience with technology at all. Of the 216 survivors who consented to participate in this study, 50.9% (110) were randomized to the UC arm, and 49.1% (106) randomized to the OSMT intervention arm. Of the 106 survivors randomized to intervention, 12.2% (13) subsequently opted not to participate in any OSMT sessions. The reasons for not joining any sessions included the following: technology concerns 31% (4/13), illness 15% (2/13), and opting out of the study 54% (7/13). Of the 106 intervention participants, 87.7% (93) attended at least 1 OSMT session. Of the 93 participants that attended OSMT sessions, 86% (80) completed the OSMT curriculum and 14% (13) did not complete the OSMT curriculum. The reasons for not completing the OSMT curriculum included technology concerns 15% (2/13), advanced illness or death 46% (6/13), and no longer wanting to participate 39% (5/13). Moreover, 21 UC participants and 28 intervention participants did not complete the study for the reason lost to follow-up. Of all participants lost to follow-up, 57% (12/21) of UC participants and 39% (11/28) of intervention participants were lost to follow-up at 6 months.

### Telehealth Technology–Related Incidents

OSMT-session field notes were logged for all 102 completed group OSMT sessions. Frequencies of noted TTR observations are presented in Table 2.

Occurrences of TTR incidents described in the OSMT-session field notes were divided into two categories: major and minor incidents (Table 3). Major incidents included a telehealth engineer intervening to provide technical support such as working one-on-one with a participant, offline from the session in-progress in some instances, to resolve a range of problems such as lack of audio or poor audio quality, camera placement or image quality, or network performance to enable the participant to rejoin the group. Minor incidents did not require a telehealth engineer intervening. Some examples of minor incidents include peers or nurse educators assisting participants with microphone muting and unmuting controls, a participant disconnecting and then reconnecting to the session on their own, or a participant experiencing a minor degradation of audio or video. Of the 102 completed OSMT sessions, 36.3% (37) had at least 1 TTR incident noted (Table 3). No session had more than 2 TTR incidents noted.

**Table 2:** Frequencies of Ostomy Self-management Telehealth sessions with 0, 1, or 2 telehealth technology–related incidents noted per session (n=102).

TTR^a^ incidents noted per session	Values, n (%)
0 incidents	65 (63.7)
1 incident	28 (27.5)
2 incidents	9 (8.8)

^a^TTR: telehealth technology–related.

**Table 3:** Frequency distribution of major and minor telehealth technology–related (TTR) incidents noted in 37 sessions that had at least one TTR incident.

Type of telehealth technology–related incident noted	Sessions, n (%)
2 major and 0 minor	4 (11)
1 major and 1 minor	3 (8)
1 major and 0 minor	15 (40)
2 minor and 0 major	2 (5)
1 minor and 0 major	13 (35)

No OSMT sessions were canceled or rescheduled because of TTR failure. A total of 3 sessions were canceled for reasons unrelated to telehealth technology.

A total of 46 TTR incidents, occurring during a total of 37 OSMT sessions, were noted. In total, 21.5% (22/102) sessions had a major incident. From the 46 TTR incidents, audio and internet connection problems accounted for most TTR incidents with a combined total of 36 (78%) incidents, whereas problems with video, software, and equipment totaled 6 (13%) incidents. Furthermore, 9% (4/46) of problems noted did not specify the type of technology problem encountered.

Most TTR incidents were experienced by the ostomates, as compared with the peers or the nurse educators. While 28 noted incidents described technology problems primarily experienced by ostomates (n=26) and caregivers (n=2), 16 technology problems were experienced by peers (n=11), nurse educators (n=3), or both peers and nurse educators (n=2). Two noted incidents did not specify participants who experienced the technology problem.

### Telehealth OSMT Intervention Receptivity

The OSMT program was generally well-received, and many intervention participants emphasized the ease and accessibility of the OSMT program provided by the telehealth component. One peer noted, after a particularly upbeat session, how “superior” the telehealth format was compared to in-person support groups because of the “ease in format for meeting and communicating with people who live apart.” Comments from peers and ostomates highlighted important aspects of the secure telehealth real-time videoconference format for the OSMT sessions, citing the “safe environment for expressions of vulnerability and important emotions” and the reliability of the telehealth platform to “function effectively despite severe weather,” which would have discouraged travel to an in-person meeting.

## Discussion

### Principal Findings

The results of this study are timely owing to the dramatic surge in telehealth usage propelled by the COVID-19 pandemic. The ATP’s telehealth engineers are experienced in supporting and testing complex telemedicine networks in demanding environments such as rural and prison environments with unstable access to broadband telecommunications and challenging cancer clinical patient-training settings such as the multisite SGVC cancer survivor support training sessions for ostomates, as described in this paper.

Multiple patient participants in SGVCs can potentially increase the probability of session interruptions due to TTR incidents. On the other hand, multiple participants in each session are also desirable for enriching the learning experience for the patient participants and increasing the number of patients who complete the training program. The probability of interruption or failure to complete an OSMT training session for TTR reasons becomes a factor in calculating the level of engineering support needed to approach zero disruptions due to TTR reasons and the cost-effectiveness of this intervention.

The ATP’s Clinical Research Unit specializes in implementing and operating high-quality cloud computing, enabling patient encounter environments. In our experience, the videoconferencing platform used in this study was robust and reliable throughout the OSMT sessions. However, there are numerous well-recognized potential technical failure points that can be barriers to successful participation in SGVCs, ranging from problems with hardware, software, and network connectivity to end-user training. For example, generally, all SGVC participants are expected to install videoconferencing software on a compatible device, work with telehealth engineers to troubleshoot any TTR problems encountered when connecting to the videoconferencing platform, learn to appropriately use the microphone mute and unmute controls to prevent unintended audio disruption of the OSMT session, and learn to *produce and manage* their video image on camera.

This study designed and tested a novel telehealth engineering support strategy for a cloud computing–based videoconferencing clinical health care delivery model with a trimodal support model consisting of a combination of IPPs and IRPs: (1) proactive end-user onboarding and training to use their respective technology suite to participate in OSMT SGVC sessions; (2) telehealth engineer real-time in-session monitoring, management, and support of OSMT-session participants, including patients, peers, and nurse educators; and (3) proactive telehealth engineer follow-up technical support and problem-solving to address and resolve any TTR problems not resolved during an OSMT session. In this randomized controlled patient clinical trial, despite the occurrence of TTR incidents in some OSMT sessions, no sessions were canceled due to TTR failure. This high OSMT-session operational success level was achieved by (1) incorporating 15-90 minutes of presession, one-on-one patient-telehealth engineer instruction sessions; (2) assigning an in-the-classroom dedicated telehealth engineer to *all* sessions; and (3) including nurse educators, site coordinators, and peers on the technology assistance team tasked with minor technology problem resolution during the SGVCs, whereas the in-the-classroom telehealth engineer handled both major and minor technical problems encountered by OSMT participants.

OSMT for cancer survivors held via SGVCs, as described herein, involves multiple new ostomates, multiple peers, 1 or 2 nurse educators, and a dedicated telehealth engineer in each training session. The justification for inclusion of the dedicated telehealth engineer was based on our previous experience that disruptions in sessions may have serious negative ramifications for participants. Disruptions of sessions due to technological difficulties can elevate fear and doubt of technology in patients and result in the accumulation of nonproductive time for training group members. Technology issues are of potential concern for multiuser cloud computer-based interventions for cancer support groups [16,17,19-21]. This study was designed to determine the frequency with which telehealth engineer support was needed to address technical failures in OSMT sessions. Over the duration of the OSMT study, no videoconferencing service platform outages disrupted OSMT sessions. There are rare instances when the videoconferencing service platform is known to have experienced an outage or reduction in performance [22]. In this study, participants experienced a range of TTR incidents. Problem areas included establishing sufficient network connectivity for some participants, instructing participants on technology configuration and operations, participants remembering to *mute* or *unmute* their microphones or to enable or disable their device camera, and participants achieving sufficient illumination to allow others on the SGVC to see them.

In anticipation of potential technical incidents affecting patients during SGVC sessions (eg, loss of audio or video camera malfunction), redundancy of personnel was intentionally built into the protocol for both the nurse educator components and the telehealth technology support components of the OSMT sessions. To accomplish this, 2 fully prepared nurse educators were present in the videoconference room used by a pair of nurse educators to lead OSMT groups in 94.1% (96/102) of the sessions. The assigned telehealth engineer was stationed in the videoconference room, rather than being at his usual site of operations, a nearby videoconference control room. Nurse educators became a part of the onsite technology team. They were trained to step in and resolve minor technology issues when the telehealth engineer was occupied with other support tasks. Third, planned redundancy was incorporated into the OSMT curriculum so that each ostomate could have full exposure to the curriculum by attending three out of four training sessions. These measures proved to be effective in minimizing interruptions of OSMT sessions for technical reasons and maximizing OSMT curriculum completion by ostomates.

Currently, rapid changes are occurring in the telemedicine industry [13]. Use of telemedicine (health care services by a physician at a distance) and telehealth (health care services delivered by nonphysicians, eg, nurses, pharmacists, or psychologists) is skyrocketing. Much of this is attributed to the COVID-19 pandemic and the *Stay at Home* mandates of US federal and state governments. It is estimated that there will be close to a billion telehealth cases in the United States in 2020 alone, up from 36 million cases in 2019 [13]. This surge in activity invites more studies on the many facets of telemedicine and telehealth service delivery, bringing quality of services, cost-effectiveness of telehealth, clinical outcomes, user satisfaction, and the applicability of videoconferencing services, into sharper focus [15-17,19].

This study examined questions related to the technological aspects of telehealth-enabled patient training. For example, how can ostomy patients be suitably prepared for support group training sessions? What level of technical support can achieve a zero level of session failure in a cancer survivor group training activity?

The results of this study also provide baseline data on the range of video communication devices used by today’s patients enrolled in a community-based OSMT cancer patient–training group setting.

### Limitations

One limitation of this study was that recorded TTR incidents comprised free-text comments from a group of individuals with varying levels of technology expertise. Another limitation was that the notes describing TTR incidents generally did not include measures of time spent on either incident resolution or disruptive impact to OSMT sessions. Currently, in a separate study, we are following a more structured protocol with regard to observing and recording TTR incidents.

### Conclusions

The delivery of OSMT via SGVCs can enable interactive OSMT for groups of new ostomates from anywhere they have access to broadband internet and sufficient privacy. The technical staffing model and combination of IPPs and IRPs for OSMT described in this paper worked to minimize session disruptions for TTR reasons. Additional research is needed to determine the scalability of OSMT for larger groups across multiple time zones.

## Abbreviations

ATP: Arizona Telemedicine Program
CONSORT: Consolidated Standards of Reporting Trials
FC: family caregiver
IPP: incident prevention plan
IRP: incident response plan
OSMT: Ostomy Self-management Telehealth
SGVC: secure group video call
SP: support person
TTR: telehealth technology–related
UC: usual care
WOCN: wound, ostomy, and continence nurse
